# Supplementary material for: Cysteines 1078 and 2991 cross-linking plays a critical role in redox regulation of cardiac ryanodine receptor (RyR)
Source: Nat Commun. 2023 Jul 26;14:4498. doi: 10.1038/s41467-023-40268-z (PMC10372021; doi:10.1038/s41467-023-40268-z)
Supplement: Supplementary file 1 — Supplementary Information [file 41467_2023_40268_MOESM1_ESM.pdf]

SUPPLEMENTARY INFORMATION

"Cysteines 1078 and 2991 cross-linking plays a critical role in redox regulation of cardiac RyR"

Supplementary Figure 1

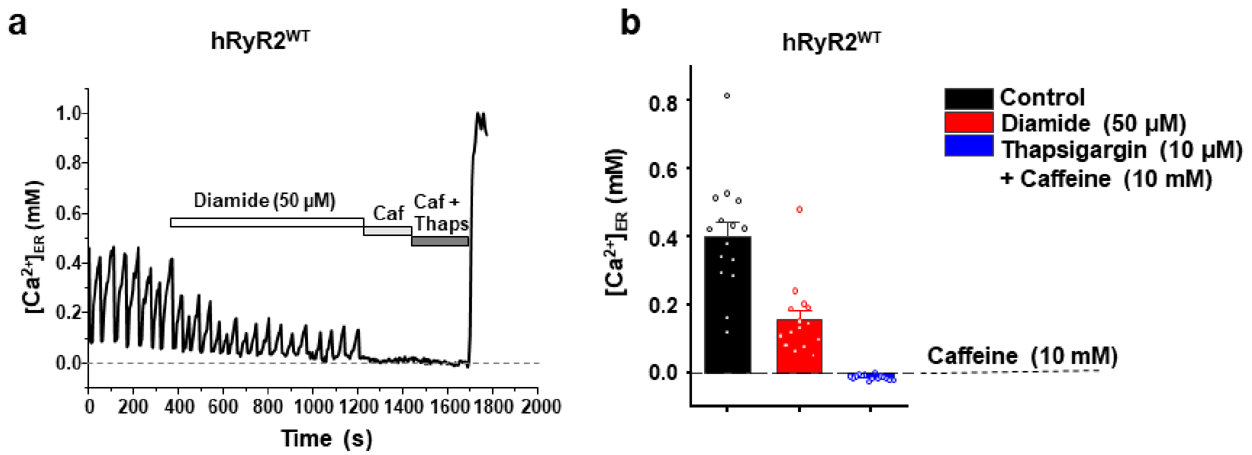

The caffeine application causes complete ER Ca^{2+} depletion in HEK293 cells expressing hRyR2. **a.** Representative recordings of Ca^{2+} waves in HEK293 cells expressing hRyR2^{WT} and SERCA2a. Diamide (50 μM) was added to induce hRyR2^{WT} oxidation. Caffeine (Caf; 10 mM) was applied to completely deplete $[Ca^{2+}]_{ER}$. F_{max} was estimated by applying ionomycin (2 μM), escin (0.005%), and 10 mM Ca^{2+}. Thapsigargin (Thaps; 10 μM) was applied to confirm complete depletion $[Ca^{2+}]_{ER}$ by caffeine. **b.** Normalized levels of ER Ca^{2+} load after application of diamide and thapsigargin. The dashed line represents a level of $[Ca^{2+}]_{ER}$ in the presence of caffeine (10 mM) alone. Data are shown as means ± SE (n = 15 cells).

Supplementary Figure 2

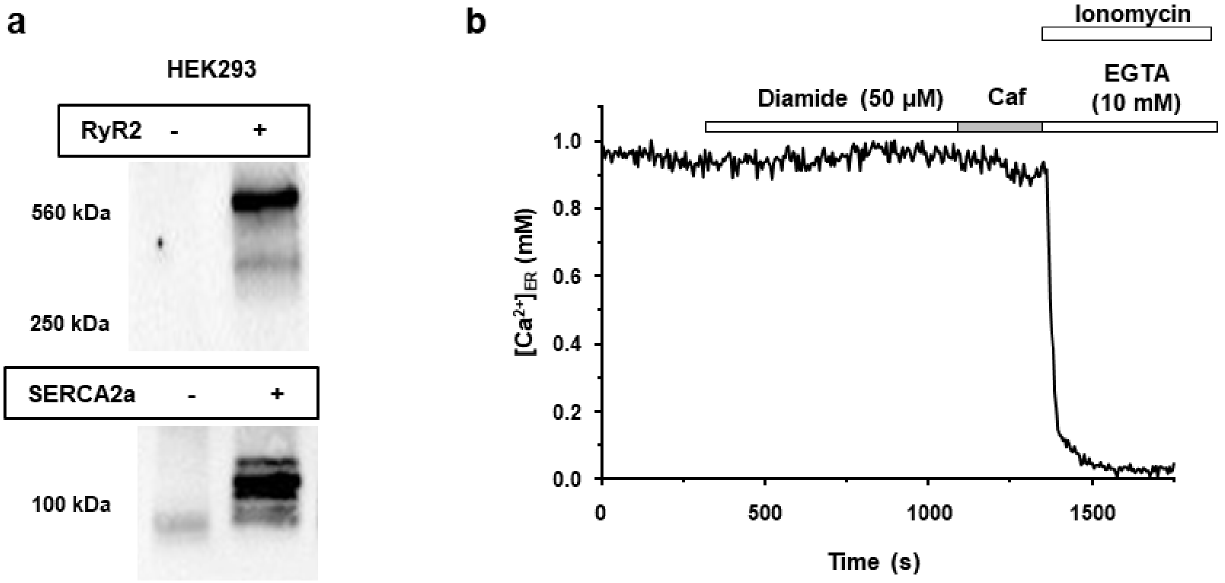

The diamide concentration that causes the maximal RyR2 cross-linking does not affect ER Ca^{2+} leak/load in cells that do not express hRyR2. a. Western blot image of HEK293 cells ("-" no transfection, "+' transfected with mCer-SERCA2a and GFP-hRyR2 plasmids). HEK293 cells express endogenous SERCA but not RyR. Data are representative of 3 independent experiments. **b.** Representative recordings of $[Ca^{2+}]_{ER}$ from Flp-In T-Rex-293 SERCA2a stable line cells expressing SERCA2a and R-CEPIA1er but not hRyR2, treated with diamide and caffeine (Caf; 10 mM). Escin (0.005%) and ionomycin (2 μM) in 10 mM EGTA (F_{min}) or 10 mM Ca^{2+} (F_{max}) were used to normalize the signal.

Supplementary Figure 3

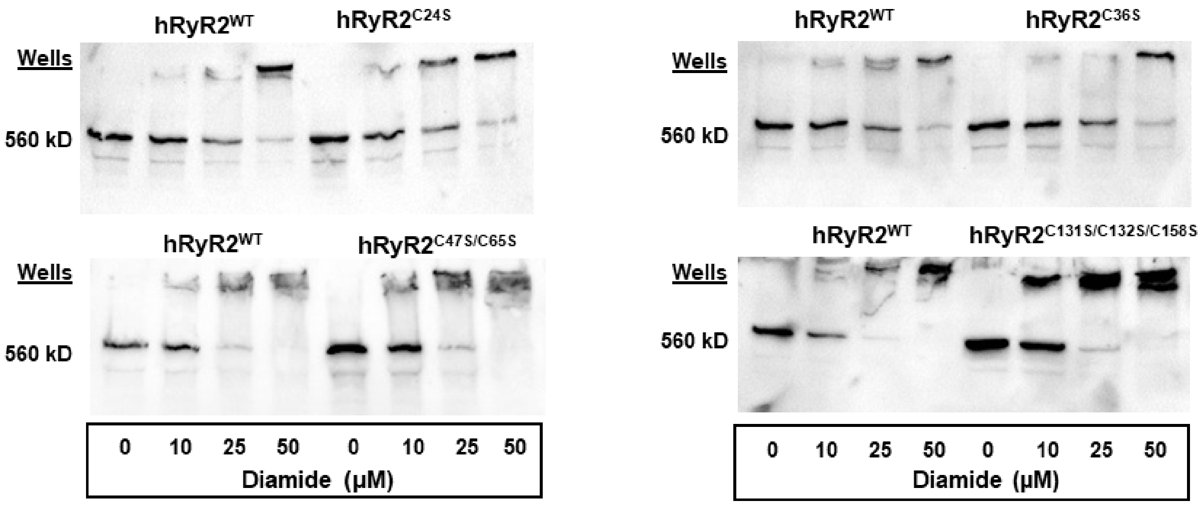

Cysteine residues from hRyR2 N-terminal domain A do not provide thiol groups for intersubunit disulfide cross-linking. Western blot images of diamide-induced hRyR2 cross-linking in HEK293 cells expressing the $hRyR2^{WT}$ and hRyR2 mutants with mutations of cysteines localized in N-terminal domain A ($hRyR2^{C24S}$, $hRyR2^{C36S}$, $hRyR2^{C47S/C65S}$, $hRyR2^{C131S/C132S/C158S}$). All data are representative of 3 independent experiments.

Supplementary Figure 4

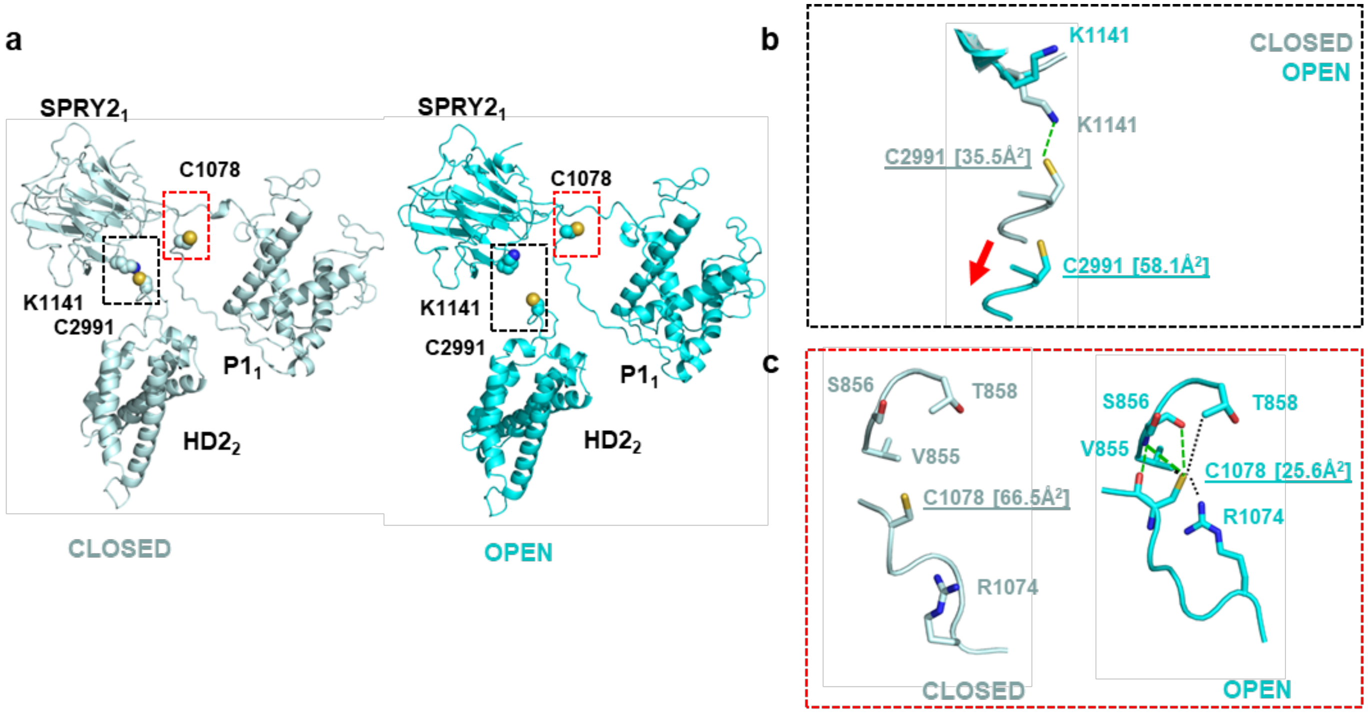

Graphical representation of the molecular environment of cross-linking cysteines 1078 and 2991 in closed and open conformations of hRyR2^{WT}. **a.** View of P1, SPRY2 and HD2 domains of hRyR2 in closed and open conformations (PDB IDs: 7UA5, 7UA9). Subscript indexes represent RyR2 subunit 1 or 2. **b.** A zoomed-in area of the C2991 environment in hRyR2 open and closed conformations. **c.** A zoomed-in area of the C1078 environment in hRyR2 open and closed conformations.

The structures of closed and open dephosphorylated RyR2 with no CaM (PDB IDs: 7UA5; 7UA9) were analyzed first. C2991 is located at the loop of the HD2 domain close to the P1 – SPRY2 linker that includes C1078 (Fig 4a). In the closed conformation, the SH-group of C2991 is oriented towards the SPRY2 domain in a way that makes a hydrogen bond with K1141 (Fig 4a and b). This hydrogen bond contributes to the decreased but relatively high ASA of 35.5 Å^2. This value improves up to 58.1 Å^2 during the RyR2 transition to the open state (Fig 4b). This effect could result from the change in intersubunit distances between the SPRY2 domain on the first subunit and the HD2 domain on the second one. Consequently, the distance between C2991 and K1141 increases, eliminating the ability to form a hydrogen bond. The accessibility of cross-linking C1078 has a high ASA of 66.5 Å^2 in the closed conformation and decreased ASA of 25.6 Å^2 in the open conformation (Fig 4c). The drop in ASA is mainly associated with the C1078 interactions with residues of the SPRY1-P1 linker (V855, S856, and T858) and neighboring R1074 on the same subunit. Due to limited local resolution in this area, a correlation between the RyR2 conformations and the C1078 accessibility to oxidation cannot be reliably established. The P1-SPRY2 linker could have a high level of flexibility that may lead to C1078 thiol group exposure, at least in some conformations.

For all structural figures (Figures 4, 7-10). *P1 and SPRY2 domains of corresponding cryo-EM models were superposed (WinCoot 0.9.8.1, LSQ, C-alphas) to compare the relative changes in the residues' environment. Dashed green lines between residues' side chains indicate hydrogen bonds, and dashed black lines indicate van der Waals interactions. Thiol groups' ASA values are provided in the square brackets.*

Supplementary Figure 5

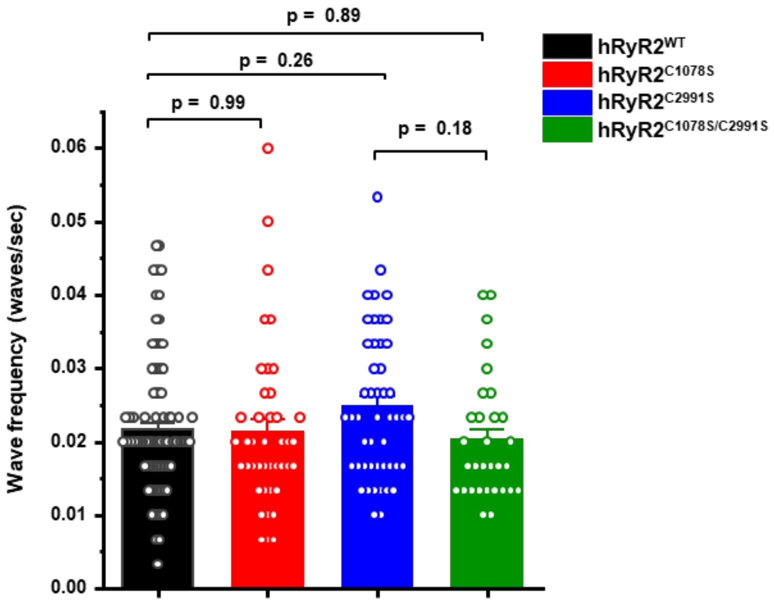

Cysteine mutants and hRyR2^{WT} generate Ca^{2+} waves with similar properties. Frequencies of Ca^{2+} waves generated in intact Flp-In T-Rex-293 SERCA2a stable line cells expressing hRyR2^{WT} and RyR2 cysteine mutants. Data are shown as means ± SE (hRyR2^{WT}, n=101 cells; hRyR2^{C1078S}, n=42 cells, hRyR2^{C2991S}, n=48 cells; hRyR2$^{C1078S/C2991S}$, n=31 cells). Data were analyzed by one-way ANOVA followed by Tukey's post-hoc test.

Supplementary Figure 6

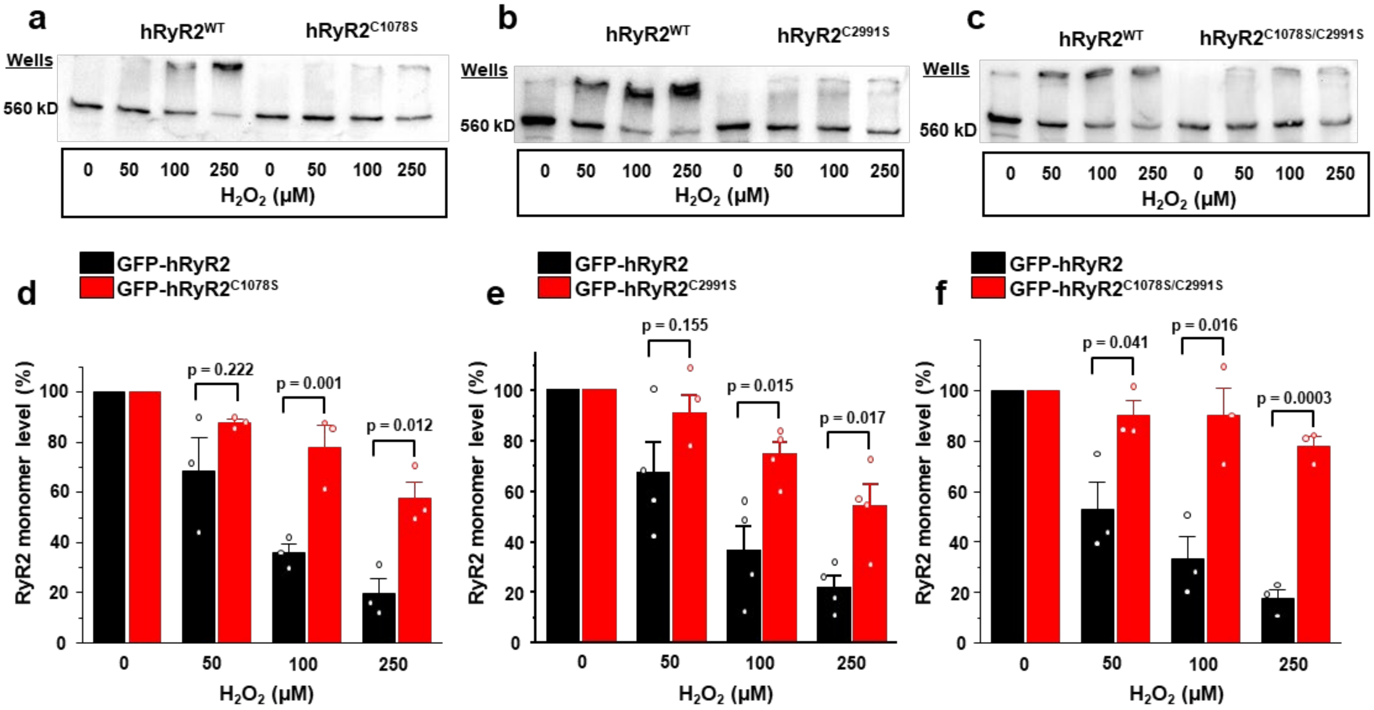

The mutation of cysteines 1078 and 2991 to serines prevents disulfide cross-linking of hRyR2 during oxidation by H_2O_2. a-c. Representative western blot images of cross-linking in HEK293 cells expressing hRyR2^{WT}, hRyR2^{C1078S}, hRyR2^{C2991S,} and hRyR2$^{C1078S/C2991S}$ constructs. **d-f.** Summary of the effect of the increasing H_2O_2 concentrations on the level of RyR2 monomeric form for hRyR2^{WT} and hRyR2^{C1078S} (n=3), hRyR2^{C2991S} (n=4), hRyR2$^{C1078S/C2991S}$ (n=3) channels expressed in HEK293 cells. Each n represents data from an independent experiment. Data are shown as means ± SE and were analyzed using a two-sided unpaired t-test.

Supplementary Table 1

Calculated solvent-accessible surface areas (ASA) and relative solvent accessibility (RSA) for cross-linking cysteines 2991 and 1078 and their thiol groups. The cryo-EM models of human hRyR2 published by *Miotto et al.* were used for the analysis[1]. Each number represents an average value derived from the analysis of four subunits in each model. The areas were calculated using the AREAIMOL program from CCP4i package (v. 8.0.007)[2]. For reference, the estimated maximal ASA for the cysteine's thiol group is 82.07 Å^2 as measured in the Glycine-Cysteine-Glycine tripeptide[3]. Based on this value, RSA was calculated to estimate the relative exposure of SH-groups. For entire residues, those with RSA values of more than 20% are considered exposed to solvent[4].

**PDB-ID**	**Gating State**	**-PO_4**	**WT/ Mutant**	**CaM**	**ARM210**	**C2991 (per SH-group)**		**C2991 (per residue)**		**C1078 (per SH-group)**		**C1078 (per residue)**	
						**ASA (Å^2)**	**RSA**	**ASA (Å^2)**	**RSA**	**ASA (Å^2)**	**RSA**	**ASA (Å^2)**	**RSA**
7UA5	***Closed***	no	**WT**	no	no	35.48	0.43	88.98	0.60	66.45	0.81	113.30	0.82
7UA9	***Open***	no	**WT**	no	no	58.08	0.71	85.80	0.59	25.60	0.31	33.93	0.26
7U9T	***Closed***	**yes**	**WT**	**yes**	no	58.85	0.72	102.55	0.71	40.28	0.49	49.23	0.36
7U9Q	***Closed***	**yes**	**WT**	no	no	45.85	0.56	73.00	0.49	32.35	0.39	40.55	0.31
7U9R	***Open***	**yes**	**WT**	no	no	63.35	0.77	98.75	0.69	26.03	0.32	38.73	0.29
7UA3	***Closed***	**yes**	**R2474S**	**yes**	no	48.25	0.59	116.78	0.83	43.95	0.54	50.90	0.38
7UA1	***Closed***	**yes**	**R2474S**	no	**yes**	57.15	0.70	96.48	0.67	45.38	0.55	61.83	0.45
7U9X	***Closed***	**yes**	**R2474S**	no	no	43.55	0.53	93.15	0.66	19.85	0.24	32.50	0.24
7UA4	***Open***	**yes**	**R2474S**	**yes**	no	55.75	0.68	117.98	0.79	52.45	0.64	65.55	0.45
7U9Z	***Open***	**yes**	**R2474S**	no	no	29.43	0.36	36.45	0.27	39.65	0.48	51.20	0.37

Supplementary Figure 7

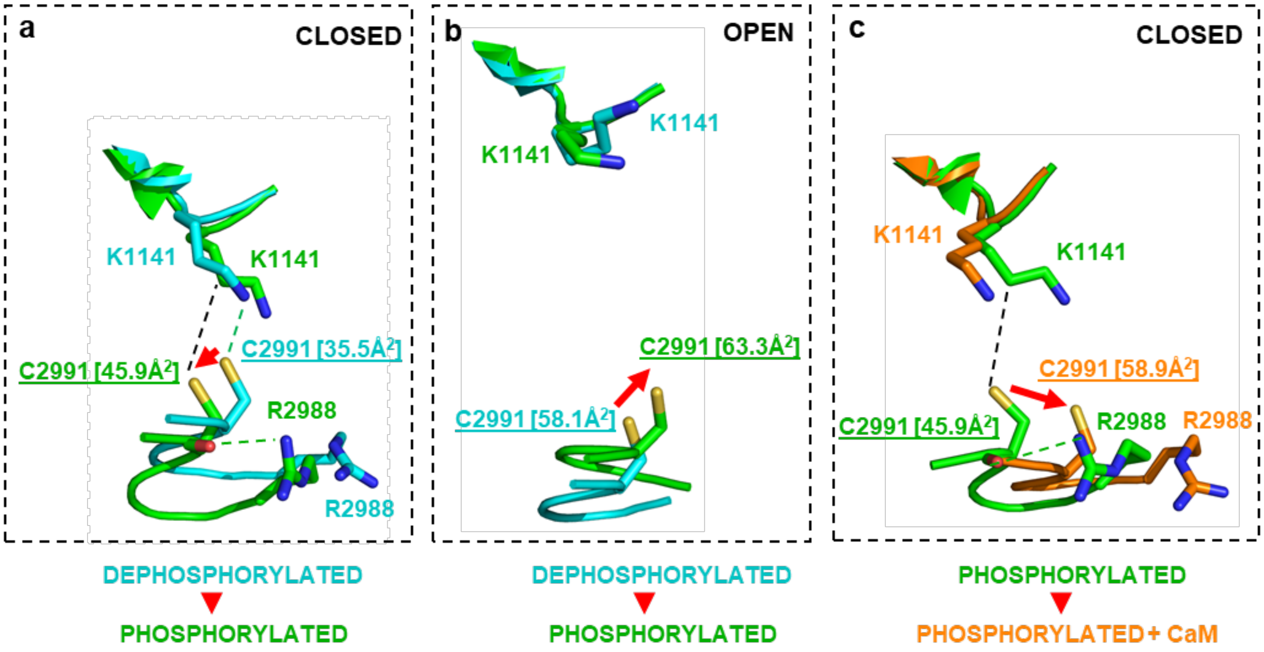

A zoomed-in view of the molecular environment of cross-linking cysteine 2991 of hRyR2^{WT} in different conformations: the effect of phosphorylation and CaM binding.

a. Comparison of dephosphorylated and phosphorylated hRyR2 models in the closed conformation (PDB IDs: 7UA5, 7U9Q). **b.** Comparison of dephosphorylated and phosphorylated hRyR2 models in the open conformation (PDB IDs: 7UA9, 7U9R). **c.** Comparison of phosphorylated hRyR2 models with and without bound CaM in the closed conformation (PDB IDs: 7U9Q, 7U9T).

The comparison of dephosphorylated and phosphorylated models of hRyR2^{WT} (PDB IDs: 7UA5; 7UA9; 7U9Q; 7U9R) showed that PKA phosphorylation increased C2991 thiol group ASA values in both conformations from 35.5 to 45.9 Å^2 in the closed (Fig 7a) and from 58.1 to 63.3 Å^2 in the open conformation (Fig 7b). Thus, PKA phosphorylation of hRyR2 would promote exposure of the thiol group of C2991 to a solvent at both conformations. However, the open conformation of hRyR2 has a higher potential for C2991 oxidation due to higher thiol groups' ASA values and a larger gap between subunits that is more accessible for the bulkier molecules of an oxidant, such as diamide. For phosphorylated hRyR2 in the closed conformation, the binding of CaM caused a noticeable increase in the ASA value of the C2991 thiol group from 45.9 to 58.9 Å^2 (Fig 7c).

Supplementary Figure 8

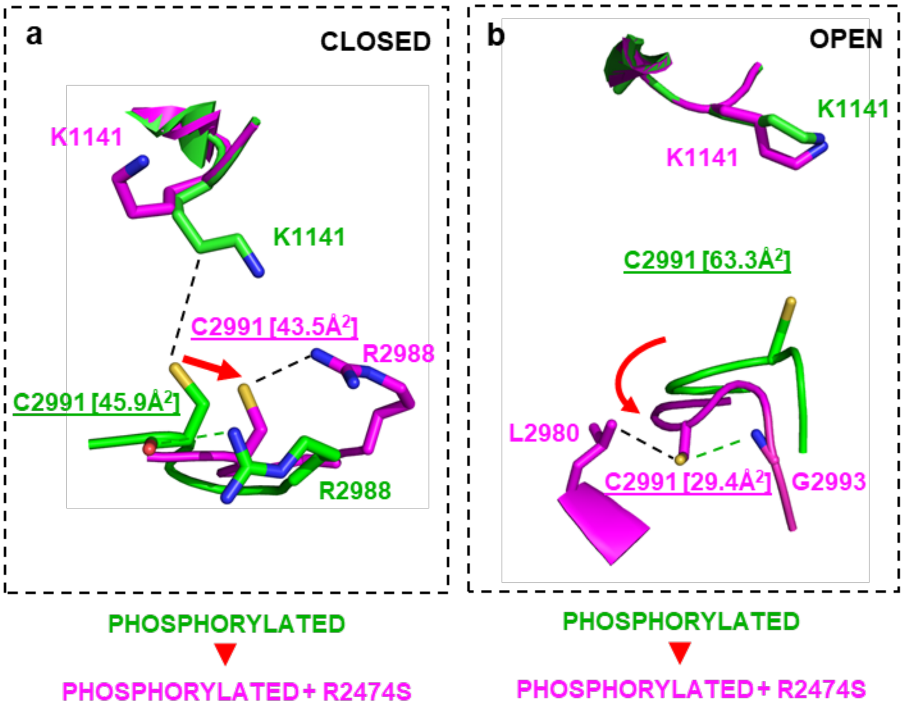

A zoomed-in view of the molecular environment of cross-linking cysteine 2991 in different conformations of phosphorylated hRyR2: the effect of R2474S CPVT mutation. a. Comparison of phosphorylated wild-type and R2474S RyR2 models in the closed conformation (PDB IDs: 7U9Q, 7U9X). **b.** Comparison of phosphorylated wild-type and R2474S RyR2 models in the open conformation (PDB IDs: 7U9R, 7U9Z).

For the available Cryo-EM models of phosphorylated RyR2, an introduction of the point R2474S CPVT mutation did not substantially change the C2991 thiol group ASA value in the closed conformation (Fig 8a), but drastically decreased it in the open conformation from 63.3 to 29.4 Å^2 (Fig 8b). This effect was achieved by the reorientation of the C2991 thiol group inside the loop stabilized by the interactions with L2980 (van der Waals interaction) and the G2993 main chain nitrogen atom (hydrogen bond).

Supplementary Figure 9

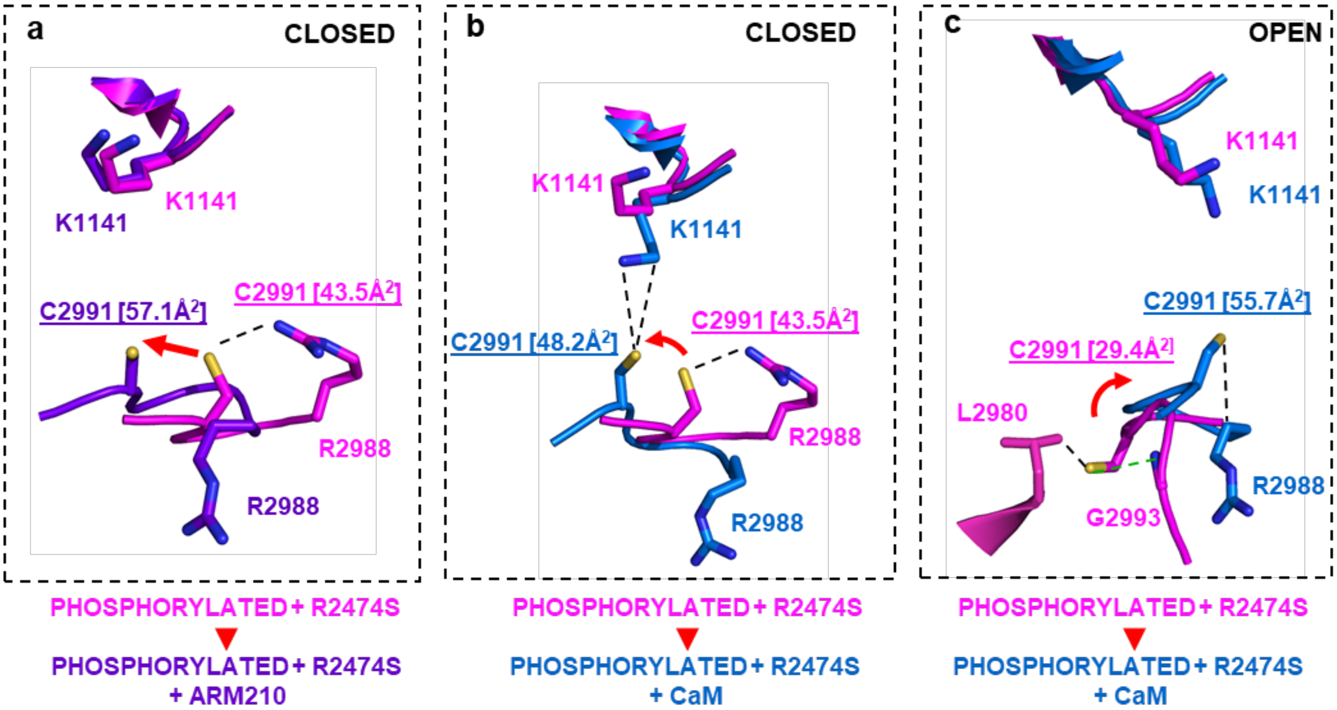

A zoomed-in view of the molecular environment of cross-linking cysteine 2991 in different conformations of phosphorylated R2474S mutant RyR2: the effect of CaM and ARM210 binding. **a.** Comparison of phosphorylated R2474S RyR2 models with and without bound ARM210 in the closed conformation (PDB IDs: 7U9X, 7UA1). **b.** Comparison of phosphorylated R2474S RyR2 models with and without bound CaM in the closed conformation (PDB IDs: 7U9X, 7UA3). **c.** Comparison of phosphorylated R2474S RyR2 models with and without bound CaM in the open conformation (PDB IDs: 7U9Z, 7UA4).

The presence of stabilizing agent ARM210[1] led to an increase of the C2991 thiol group ASA value in the phosphorylated C2474S mutant in the closed conformation from 43.5 to 57.1 Å^2 (Fig 9a). CaM binding in the same structure had a smaller effect and increased area to 48.2 Å^2 (Fig 9b). CaM binding to the C2474S mutant at the open conformation was able to reorient the C2991 thiol group outwards, restoring its ASA value from 29.4 to 55.7 Å^2.

Supplementary Figure 10

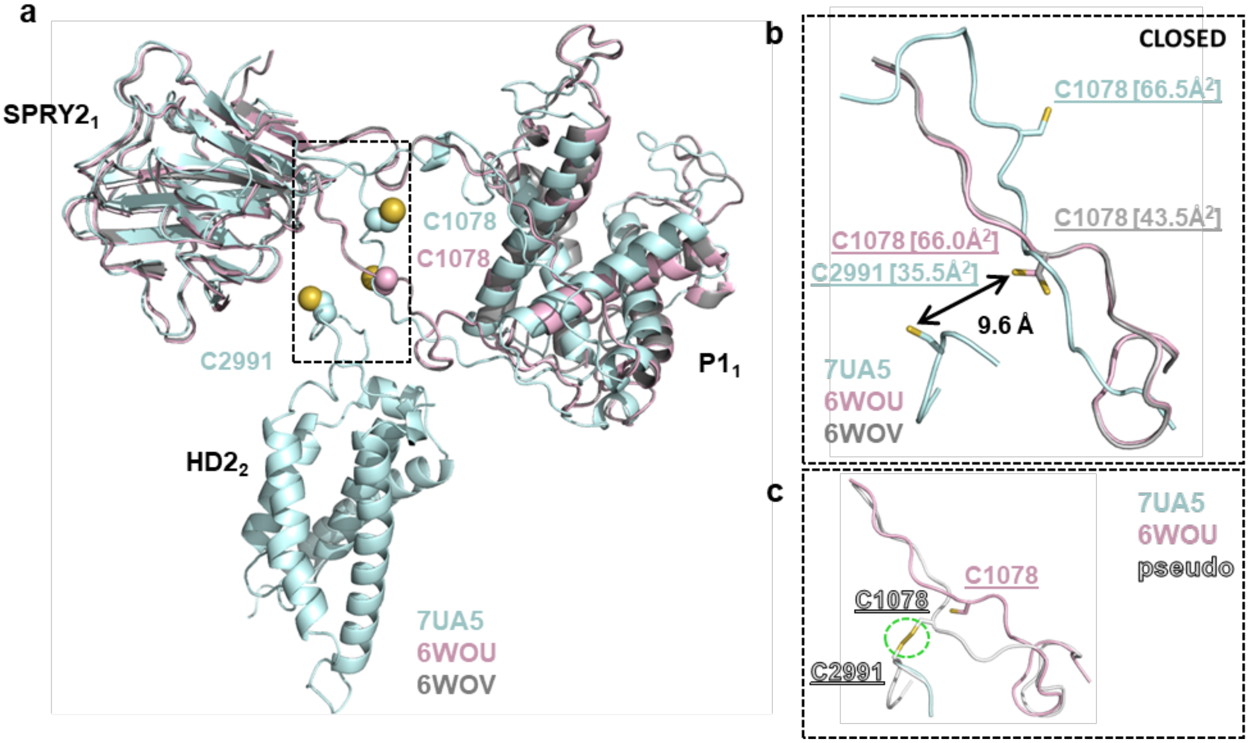

A comparison of P1-SPRY2 linker conformation from RyR2 Cryo-EM models obtained from different groups. a. View of P1, SPRY2, and HD2 domains of WT RyR2 in closed conformation obtained by Miotto et al. (PDB ID: 7UA5) superposed with the same domains from the models of WT and R176Q RyR2 models obtained by Iyer et al. (PDB IDs: 6WOV, 6WOU). Subscript indexes represent RyR2 subunit 1 or 2. **b.** A zoomed-in view of different RyR2 P1-SPRY2 linker conformations and cross-linking cysteines' orientations in different models. **c**. Manual modeling of pseudo conformation of RyR2 P1-SPRY2 linker compatible with cross-linking of cysteines 1078 and 2991 (WinCoot 0.9.8.1) based on the existing cryo-EM models of RyR2.

Available Cryo-EM structures[1] provide important information about the orientation of C2991, whereas the exact conformation of C1078 on the flexible linker seems unclear due to limited resolution in this area. To investigate possible conformations of the P1-SPRY2 linker and specifically C1078, we compared the cryo-EM models of the closed $RyR2^{WT}$ at resolution 2.83 Å (PDB ID: 7UA5)[1] and two structures of closed $RyR2^{WT}$ and $RyR2^{R176Q}$ (PDB IDs: 6WOV, 6WOU; resolutions 5.1 and 3.27 Å)[5]. Despite the CPVT mutation in the 6WOU model, both WT and R176Q mutant RyR2 share a similar P1-SPRY2 linker conformation. At the same time, the mutant model has a substantially better resolution (3.27 Å), which makes it an overall more reliable source of structural information. The position of another cross-linking cysteine (C2991) was derived from the 7UA5 model, which has a better resolution in this area.

The superposition (WinCoot 0.9.8.1, LSQ, Cα) of the corresponding SPRY2 and P1 domains (Fig 10a and b) showed that depending on the linker conformation, C1078 could be oriented towards C2991 (PDB IDs: 6WOV; 6WOU) or in the opposite direction (PDB ID: 7UA5). Both linker conformations seem to be geometrically allowed, given that all models were validated before the deposition to the PDB bank. Using the linker conformation from model 6WOU as a template, we manually rebuilt the linker (WinCoot 0.9.8.1)

to artificially introduce a disulfide bond between C2991 and C1078 (Fig 10c). The obtained pseudo linker conformation was geometrically refined in WinCoot software to avoid the introduction of forbidden conformations (bonds, angles, rotamers). Based on this simple modeling and surface accessibility numbers, we believe C1078 may physically reach C2991 to form a disulfide bond (Fig 10c). At the same time, it appears that the P1-SPRY2 linker should be stabilized in a specific conformation to make the disulfide bond formation possible.

Supplementary Figure 11

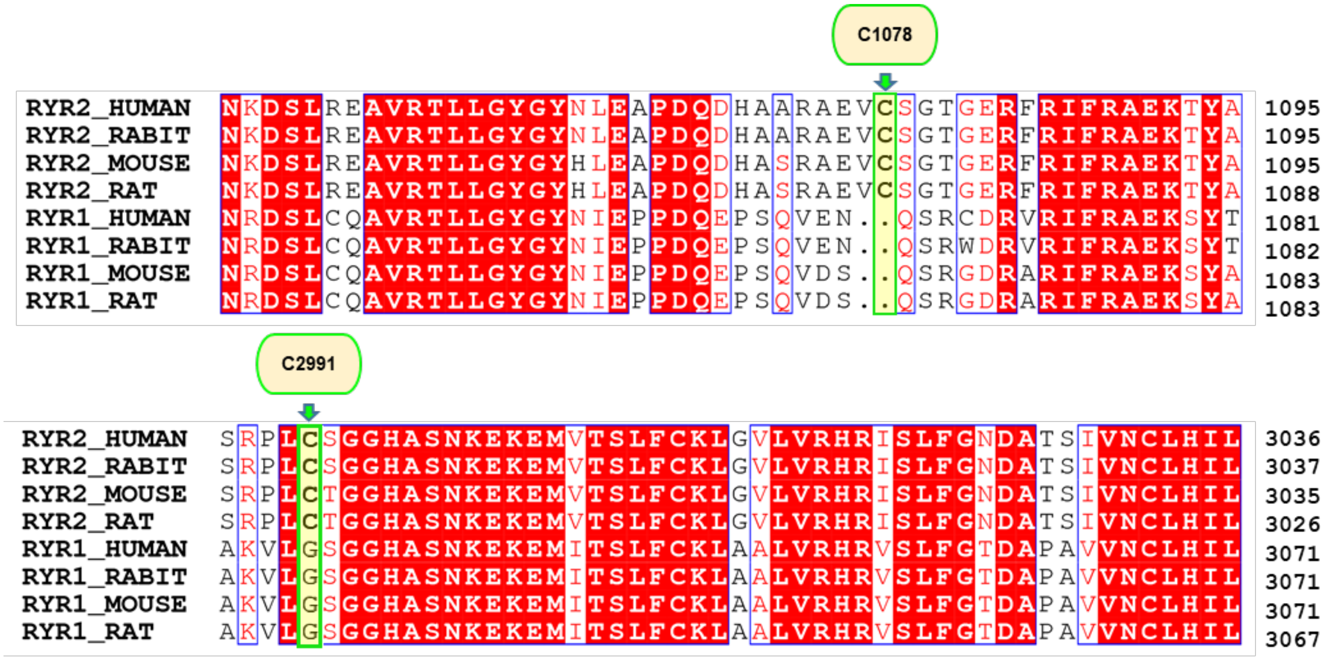

Cysteines 1078 and 2991 are conserved in mammalian RyR2 but are missing in RyR1. Visualization by ESPript 3.0[6].

Supplementary Table 2

List of oligonucleotide sequences used in the paper

	**PRIMERS**	
**Residue on RyR2**	FORWARD	REVERSE
C24S	CTGCAGAGCACCGCAACCAT CCACAA	AACCACTTCATCATCAGTTCGCA GGAACT
C36S	CAGAAGCTAAGCTTGGCAGC AGAAGGAT	TTGTTCTTTGTGGATGGTTGCGG TGC
C47S	AACAGACTTAGTTTCTTGGAG TCC	GCCAAATCCTTCTGCTGCCAAGC ATAGC
C65S	CTCTCCATCAGCACCTTTGTG CTGGAGCA	GTCTGGGGGCACATTCTTGGAAT TGG
C131S/C132S	TATCTGAGCAGCCTGTCCACC TCCCGGTCTT	CATGCCACTATAGGAATGGCGCA GCAATATGGCATGTCC
C158S	AGGCTTCTTGGTGGACCATAC ACCCTGCC	CCCCTGTGGTGTCCTCTTGCAAG CCAACATC
C1078S	AGCCGAAGTGTCCAGCGGCA CCGGGGAAAGG	CTGGCTGCATGATCTTGATCTGG TGCTTCCAAGTTGTAGCC
C2991S	AAGCAGACCTCTCaGCTCTGG AGGACATGCTTCC	GCTGCAGATAAGAAGTATAAACG ATGGTTTTG

SUPPLEMENTARY REFERENCES

1. Miotto, M. C. *et al.* Structural analyses of human ryanodine receptor type 2 channels reveal the mechanisms for sudden cardiac death and treatment. *Sci. Adv.* **8**, eabo1272 (2022).

2. Potterton, E., Briggs, P., Turkenburg, M. & Dodson, E. A graphical user interface to the CCP4 program suite. *Acta Crystallogr. D. Biol. Crystallogr.* **59**, 1131–7 (2003).

3. Samanta, U., Bahadur, R. P. & Chakrabarti, P. Quantifying the accessible surface area of protein residues in their local environment. *Protein Eng.* **15**, 659–67 (2002).

4. Savojardo, C., Manfredi, M., Martelli, P. L. & Casadio, R. Solvent Accessibility of Residues Undergoing Pathogenic Variations in Humans: From Protein Structures to Protein Sequences. *Front. Mol. Biosci.* **7**, 1–9 (2021).

5. Iyer, K. A. *et al.* Structural mechanism of two gain-of-function cardiac and skeletal RyR mutations at an equivalent site by cryo-EM. *Sci. Adv.* **6**, 1–13 (2020).

6. Robert, X. & Gouet, P. Deciphering key features in protein structures with the new ENDscript server. *Nucleic Acids Res.* **42**, W320–W324 (2014).
