# Supplementary material for: Cysteines 1078 and 2991 cross-linking plays a critical role in redox regulation of cardiac ryanodine receptor (RyR)
Source: Nat Commun. 2023 Jul 26;14:4498. doi: 10.1038/s41467-023-40268-z (PMC10372021; doi:10.1038/s41467-023-40268-z)
Supplement: Supplementary file 3 — Reporting Summary [file 41467_2023_40268_MOESM3_ESM.pdf]

nature portfolio

Corresponding author(s): Aleksey Zima

Last updated by author(s): Jun 29, 2023

Reporting Summary

Nature Portfolio wishes to improve the reproducibility of the work that we publish. This form provides structure for consistency and transparency in reporting. For further information on Nature Portfolio policies, see our Editorial Policies and the Editorial Policy Checklist.

Statistics

For all statistical analyses, confirm that the following items are present in the figure legend, table legend, main text, or Methods section.

n/a	Confirmed	
☐	☒	The exact sample size (*n*) for each experimental group/condition, given as a discrete number and unit of measurement
☐	☒	A statement on whether measurements were taken from distinct samples or whether the same sample was measured repeatedly
☐	☒	The statistical test(s) used AND whether they are one- or two-sided<br>*Only common tests should be described solely by name; describe more complex techniques in the Methods section.*
☒	☐	A description of all covariates tested
☐	☒	A description of any assumptions or corrections, such as tests of normality and adjustment for multiple comparisons
☐	☒	A full description of the statistical parameters including central tendency (e.g. means) or other basic estimates (e.g. regression coefficient) AND variation (e.g. standard deviation) or associated estimates of uncertainty (e.g. confidence intervals)
☐	☒	For null hypothesis testing, the test statistic (e.g. *F*, *t*, *r*) with confidence intervals, effect sizes, degrees of freedom and *P* value noted<br>*Give P values as exact values whenever suitable.*
☒	☐	For Bayesian analysis, information on the choice of priors and Markov chain Monte Carlo settings
☒	☐	For hierarchical and complex designs, identification of the appropriate level for tests and full reporting of outcomes
☒	☐	Estimates of effect sizes (e.g. Cohen's *d*, Pearson's *r*), indicating how they were calculated

Our web collection on statistics for biologists contains articles on many of the points above.

Software and code

Policy information about availability of computer code

Data collection	BioRad radiance LaserSharp 2000
Data analysis	ImageJ 1.52a NIH, Origin 2021b SR2 (OriginLab U.S.A.), PyMOL 1.7.0.3, CCP4i 8.0.007, Coot 0.9.8.1, ESPript 3.0

For manuscripts utilizing custom algorithms or software that are central to the research but not yet described in published literature, software must be made available to editors and reviewers. We strongly encourage code deposition in a community repository (e.g. GitHub). See the Nature Portfolio guidelines for submitting code & software for further information.

Data

Policy information about availability of data

All manuscripts must include a data availability statement. This statement should provide the following information, where applicable:

- Accession codes, unique identifiers, or web links for publicly available datasets
- A description of any restrictions on data availability
- For clinical datasets or third party data, please ensure that the statement adheres to our policy

The data supporting this article and other findings are available within the manuscript, figures, supplementary data, and from the corresponding authors upon request. The cryo-EM models of RyR2 used in this study (PDB-IDs: 7UA5, 7UA9, 7U9T, 7U9Q, 7U9R, 7UA3, 7UA1, 7U9X, 7UA4, 7U9Z, 6WOU, 6WOV) were obtained from RSCB protein data bank (www.rcsb.com). Source data are provided with this paper.

Human research participants

Policy information about studies involving human research participants and Sex and Gender in Research.

Reporting on sex and gender	N/A
Population characteristics	N/A
Recruitment	N/A
Ethics oversight	N/A

Note that full information on the approval of the study protocol must also be provided in the manuscript.

Field-specific reporting

Please select the one below that is the best fit for your research. If you are not sure, read the appropriate sections before making your selection.

☒ Life sciences ☐ Behavioural & social sciences ☐ Ecological, evolutionary & environmental sciences

For a reference copy of the document with all sections, see nature.com/documents/nr-reporting-summary-flat.pdf

Life sciences study design

All studies must disclose on these points even when the disclosure is negative.

Sample size	To ensure an appropriate study design, a power analysis (alfa=0.05) was computed using approximations of anticipated effect size and standard deviation based on previous investigations. The cell experiments were grouped in seven separated experimental protocols. Using approximations for these parameters, the Power analysis suggests that 20 cells per experimental protocol are needed to achieve a power of 0.80.
Data exclusions	No data were excluded.
Replication	All western blot data were replicated in at least three independent experiments. Confocal imaging data were replicated in at least 20 cells during at least 3 independent experiments.
Randomization	Cells from the same population were used for transfection with wild-type or mutant recombinant constructs of RyR2 to be further used in biochemical and imaging experiments. For biochemical experiments cell expressing wild-type and mutant recombinant constructs were treated and processed in parallel. For imaging experiments cells expressing either wild-type or mutant constructs were randomly selected for the measurement of calcium oscillations, treatment and analysis.
Blinding	To eliminate observer's effect, key experimental findings were confirmed in blinded experiments.

Reporting for specific materials, systems and methods

We require information from authors about some types of materials, experimental systems and methods used in many studies. Here, indicate whether each material, system or method listed is relevant to your study. If you are not sure if a list item applies to your research, read the appropriate section before selecting a response.

Materials & experimental systems

n/a	Involved in the study	
☐	☒	Antibodies
☐	☒	Eukaryotic cell lines
☒	☐	Palaeontology and archaeology
☒	☐	Animals and other organisms
☒	☐	Clinical data
☒	☐	Dual use research of concern

Methods

n/a	Involved in the study	
☒	☐	ChIP-seq
☒	☐	Flow cytometry
☒	☐	MRI-based neuroimaging

Antibodies

Antibodies used	Mouse monoclonal F-1 anti-RyR2 primary antibody (1:1000; sc-376507, Santa Cruz, U.S.A.)

Antibodies used	HRP-conjugated goat anti-mouse polyclonal secondary antibody (1:5000; 31430, Thermo Fisher Scientific , U.S.A.)<br>Anti-GFP rabbit polyclonal antibody (1:1000; A6455, Thermo Fisher Scientific, U.S.A.)<br>HRP-conjugated goat anti-rabbit polyclonal secondary antibody (1:5000, A0545, Sigma-Aldrich Co, U.S.A.)
Validation	Antibody reagents were validated by Western blot analysis to determine whether the antibody is specific, recognizing a species at the correct molecular weight without binding to additional proteins.<br><br>The mouse monoclonal F-1 anti-RyR2 primary antibody (sc-376507) was validated by the manufacturer using Western blot analysis for rat heart tissue extract as positive control (https://www.scbt.com/p/ryr-antibody-f-1). We validated the antibody in Western Blot analysis using HEK293 cells transiently transfected with RyR2, GFP-RyR2 and GFP-RyR1. For the GFP-labeled RyRs, the specificity was also confirmed by using anti-GFP A-6455 antibody in parallel Western blot experiments. We additionally validated the antibody using Western blot analysis using isolated mouse ventricular cardiomyocytes as positive control. The application of this antibody was cited in 6 publications:<br><br>1. Tsuda, T., et al. 2017. Corticotropin releasing hormone receptor 2 exacerbates chronic cardiac dysfunction. J. Exp. Med. 214: 1877-1888.<br>2. Zuppinger, C., et al. 2017. Characterization of cytoskeleton features and maturation status of cultured human iPSC-derived cardiomyocytes. Eur. J. Histochem. 61: 2763.<br>3. Meyer, P., et al. 2021. Skeletal ryanodine receptors are involved in impaired myogenic differentiation in duchenne muscular dystrophy patients. Int. J. Mol. Sci. 22: 12985.<br>4. Desai, V.G., et al. 2022. Doxorubicin-induced delayed-onset subclinical cardiotoxicity in mice. J. Appl. Toxicol. 42: 778-792.<br>5. Hopton, C., et al. 2022. Characterization of the mechanism by which a nonsense variant in RYR2 leads to disordered calcium handling. Physiol. Rep. 10: e15265.<br>6. Yuan, W., et al. 2022. Intracellular TMEM16A is necessary for myogenesis of skeletal muscle. iScience 25: 105446<br><br>Anti-GFP rabbit polyclonal antibody was validated by the manufacturer. Antibody specificity was demonstrated by detection of different targets fused to GFP tag in transiently transfected lysates tested. Relative detection of GFP tag was observed across different proteins fused with GFP in H3-GFP and p65-GFP. GFP-variant, YFP is also being detected in His-p65-YFP lysate, using Anti-GFP Polyclonal Antibody (Product # A-6455) in Western Blot (https://www.thermofisher.com/antibody/product/GFP-Antibody-Polyclonal/A-6455).

Eukaryotic cell lines

Policy information about cell lines and Sex and Gender in Research

Cell line source(s)	HEK-293 AAVpro 293T (Takara, 632273)<br>Flp-In T-Rex-293 (ThermoFisher Scientific, R780-07)
Authentication	All cell lines used in this study were obtained from commercial manufacturers and were supplied along with the corresponding certificates of analysis.
Mycoplasma contamination	Cells used in this study are routinely screened for mycoplasma using DAPI staining and PCR. Authors confirm that all tests for mycoplasma in the cells used in this study were negative.
Commonly misidentified lines<br>(See ICLAC register)	HEK-293 cells used in the experiments do not belong to the commonly misidentified cells lines. Thus, no commonly misidentified cells lines were used in this study.
